# Metastasis of mesothelioma to the maxillary gingiva

**DOI:** 10.3892/ol.2014.2273

**Published:** 2014-06-20

**Authors:** YUICHI OHNISHI, MITSUCHIKA SUGITATSU, MASAHIRO WATANABE, TOMOKO FUJII, KENJI KAKUDO

**Affiliations:** 1 Second Department of Oral and Maxillofacial Surgery, Osaka Dental University, Osaka 540-0008, Japan; 2 Department of Dentistry and Maxillofacial Surgery, Osaka Red Cross Hospital, Osaka 543-8555, Japan

**Keywords:** metastatic, malignant, mesothelioma, maxillary gingiva

## Abstract

Malignant mesothelioma predominantly arises from the serosal surfaces of the pleural or peritoneal cavity. There is currently no effective standard treatment for mesothelioma and the prognosis for patients is poor; the majority of patients with malignant mesothelioma succumb between 12 and 17 months following diagnosis. The association of all forms of malignant mesothelioma with asbestos exposure has been well documented. However, metastasis to the oral gingiva is rare, as only four cases have previously been reported; two cases of metastasis to the tongue and four cases to the jaw bone. In the current report, the case of a 62-year-old male with metastatic mesothelioma is presented. To the best of our knowledge, this is the first report regarding the metastasis of this type of neoplasm to the maxillary gingiva.

## Introduction

Malignant mesothelioma is a rare type of tumour, which is derived from mesothelial cells of the serosal surfaces of body cavities. There is currently no effective standard treatment for mesothelioma; its prognosis is poor with the majority of patients succumbing 12–17 months following diagnosis (1). In addition, resistance to all treatment modalities is a typical feature of mesothelioma (2).

The association of all forms of malignant mesothelioma with asbestos exposure has been well documented (3,4). This neoplasm is similar to a carcinoma with regard to its mode of spread, with metastases occurring predomimantly by direct invasion into adjacent tissue, however, also via lymphatic and haematogenous routes. Distant metastasis are most common with the sarcomatous variant (5) whereas, metastatic tumours of the oral cavity are rare. Only four cases of an oral gingiva metastasis from diffuse malignant pleural mesothelioma were identified in the English language literature using a PubMed search (6–9). To the best of our knowledge, this is the first report regarding the metastasis of mesothelioma to the maxillary gingiva. Patient provided written informed consent.

## Case report

**Figure 1:**
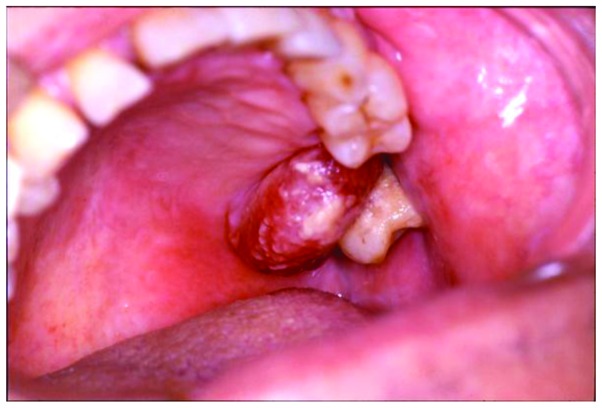
Image of the patient at initial presentation demonstrating a semi-hard, haemorrhagic lesion surrounding the molar teeth, extending to the buccal and lingual aspects of the dental alveolus.

**Figure 2:**
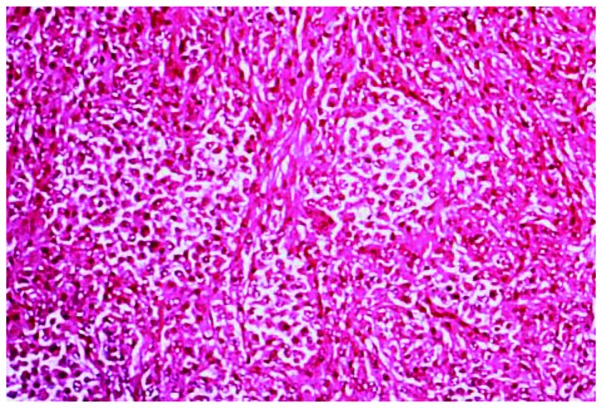
Photomicrograph demonstrating a mixed-type diffuse mesothelioma with poorly differentiated epithelial and spindle cells. (Haematoxylin and eosin stain; magnification, ×100).

**Figure 3:**
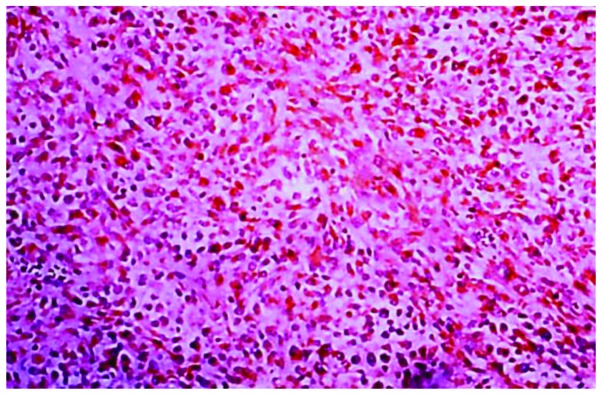
Photomicrograph immunocytochemistry identified a sarcomatous rather than a carcinomatous pattern, with strong positivity for vimentin. (Haematoxylin and eosin stain; magnification, ×100).

A 62-year-old male was referred to the physicians at the Department of Dentistry and Maxillofacial Surgery at the Osaka Red Cross Hospital (Osaka, Japan), in November 1996, with a two-month history of progressive shortness of breath on exertion. Clinical and radiographic examination revealed a left-sided pleural effusion, which was drained successfully. A chest radiograph and computed tomography demonstrated the presence of a mass in the left lung field, and a rigid bronchoscopy and open pleural biopsy showed the lesion to be a diffuse mesothelioma. At that time, the patient commenced radiotherapy (total dose, 40 Gy). However, two months after the initial presentation, the patient was referred to the Department of Dentistry and Maxillofacial Surgery, Osaka Red Cross Hospital(Osaka, Japan) with a large, painless mass on the maxillary gingiva. This mass had been present and growing gradually for two weeks. On examination a semi-hard, haemorrhagic lesion was found surrounding the molar teeth, and extending to the buccal and lingual aspects of the alveolar region (Fig. 1). The mobility of the affected teeth was good and radiographic examination revealed loss of the crestal bony architecture. An incisional biopsy was performed under local anaesthetic, and histology of the excised specimen showed a mixed-type diffuse mesothelioma with poorly differentiated epithelial and spindle cells (Fig. 2). The histological appearances of the biopsy specimen were identical to those that were observed in the previous pleural biopsy and immunocytochemistry indicated a sarcomatous, rather than carcinomatous, pattern with strong positivity for vimentin (Fig. 3). Focal positivity for cytokeratin, and negative reactions for carcinoembryonic antigen and epithelial membrane antigen were observed and an incisional biopsy was subsequently conducted. The tumour increased rapidly and the patient was unable to eat, thus, the tumour was removed to improve quality of life (QOL). The excised specimen revealed a solid semi-hard tissue mass (size, 30×25×20 mm), which was white-yellow in colour on the cut surface. Despite surgery, the patient succumbed 35 days later following deterioration of his medical condition.

## Discussion

Since 1960, when Wagner *et al* (3) demonstrated a high incidence of mesothelioma among employees working with asbestos in the Cape Province of South Africa, increasing attention has been paid to this type of tumour, and numerous reports and reviews of mesothelioma have appeared in the medical literature (10,11). Mesothelioma tend to present in adults, predominantly affecting individuals aged between 50 and 70 years, and occurs marginally more commonly in males than females (12). It arises from serosal surfaces and is approximately three times more common in the pleural cavity compared with the peritoneal cavity. The association between asbestos exposure and the development of mesotheliomas in industrial countries has been recognized (13). Previous investigations have hypothesized that longer amphibole fibres, rather than the shorter fibre types, harbor an increased potential to induce the formation of mesotheliomas (12). Furthermore, a prolonged latency period of 20–40 years following asbestos exposure and prior to the development of overt disease has also been recognized (13). In the present case, the patient was an industrial employee with a prolonged history of asbestos exposure (~24 years).

There are three common histological types of mesothelioma; epithelial (1), fibrous (2) and mixed (3), which demonstrates the close association between the epithelial and fibrous components. The epithelial type is the most common variant of diffuse malignant mesothelioma and is predominantly composed of flattened or cuboidal epithelioid cells, which form a tubular and papillary pattern. Mixed types of mesotheliomas appear as a mixture of epithelial and fibrous elements, resulting in a biphasic pattern, which at times may appear similar to synovial sarcoma. However, location, specific stains, immunohistochemical staining and the less cellular nature of mesotheliomas facilitate this differentiation (13). Differentiating between pleural epithelial mesotheliomas and pulmonary adenocarcinomas remains a diagnostic challenge for the pathologist. However, histochemical stains (13), electron microscopy and more recently, immunohistochemistry have been used to distinguish between the two entities. The present case was diagnosed as mixed-type by observation of positivity for vimentin and cytokeratin via immunohistochemical staining.

There is currently no effective standard treatment for mesothelioma and resistance to all treatment modalities is a typical feature (2). Therefore, the prognosis is poor and the majority of patients with diffuse mesothelioma succumb to the disease within one or two years.

Metastasis does occur, however, does so at a relatively late stage of the disease. Kannerstein and Churg (14) reported metastases in 18/50 autopsy cases. In their series the most common sites of metastasis were the regional lymph nodes, particularly those in the mediastinum, abdomen and supraclavicular region, and the liver, lung and bone marrow. Metastasis to the oral cavity is particularly rare, with only six cases previously reported (6–9). Since the primary site is not controlled, no case of treatment has ever been found in metastatic lesions. In the present study, and the majority of previous cases, the final diagnosis was determined by a biopsy alone, with no radical treatment administered. To the best of our knowledge, there is only one case available where the tumour at the primary site was removed, however, this is a case of lost to follow-up and it is unclear whether the primary site was really restrained. The treatment method for metastasis to the oral cavity has not been established and, considering the virulence of malignant mesothelioma and its poor prognosis, the treatment for metastasis must be determined via holistic assessment of the invasiveness and dysfunction of surgery, length of survival and QOL. In the present case, surgery on the primary site had to be abandoned due to the possibility of metastases (similar to the metastasis to the oral cavity).

In conclusion, as the total recovery of the patient in the present report was not anticipated, the patient’s QOL was considered and treatments were selected with the aim of improving the oral condition. This modality of treatment was considered to be effective as it enabled the patient to continue to eat until just prior to sucumbing to the disease.
